# Supplementary material for: A distinct topology of BTN3A IgV and B30.2 domains controlled by juxtamembrane regions favors optimal human γδ T cell phosphoantigen sensing
Source: Nat Commun. 2023 Nov 22;14:7617. doi: 10.1038/s41467-023-41938-8 (PMC10665462; doi:10.1038/s41467-023-41938-8)
Supplement: Supplementary file 1 — Supplementary Information [file 41467_2023_41938_MOESM1_ESM.pdf]

A distinct topology of intracellular and extracellular BTN3A heteromer domains supports phosphoantigen-mediated activation of human γδ T cells

Mohindar M. Karunakaran[1]*, Hariharan Subramanian[2,3], Yiming Jin[4], Fiyaz Mohammed[5], Brigitte Kimmel[6], Claudia Juraske[7-10], Lisa Starick[1], Anna Nöhren[1], Nora Länder[1], Carrie R. Willcox[5], Rohit Singh[4], Wolfgang W. Schamel[7-10], Viacheslav O. Nikolaev[2,3], Volker Kunzmann[6], Andrew J. Wiemer[4], Benjamin E. Willcox[5], Thomas Herrmann[1]*

*Corresponding authors

Figure legends:

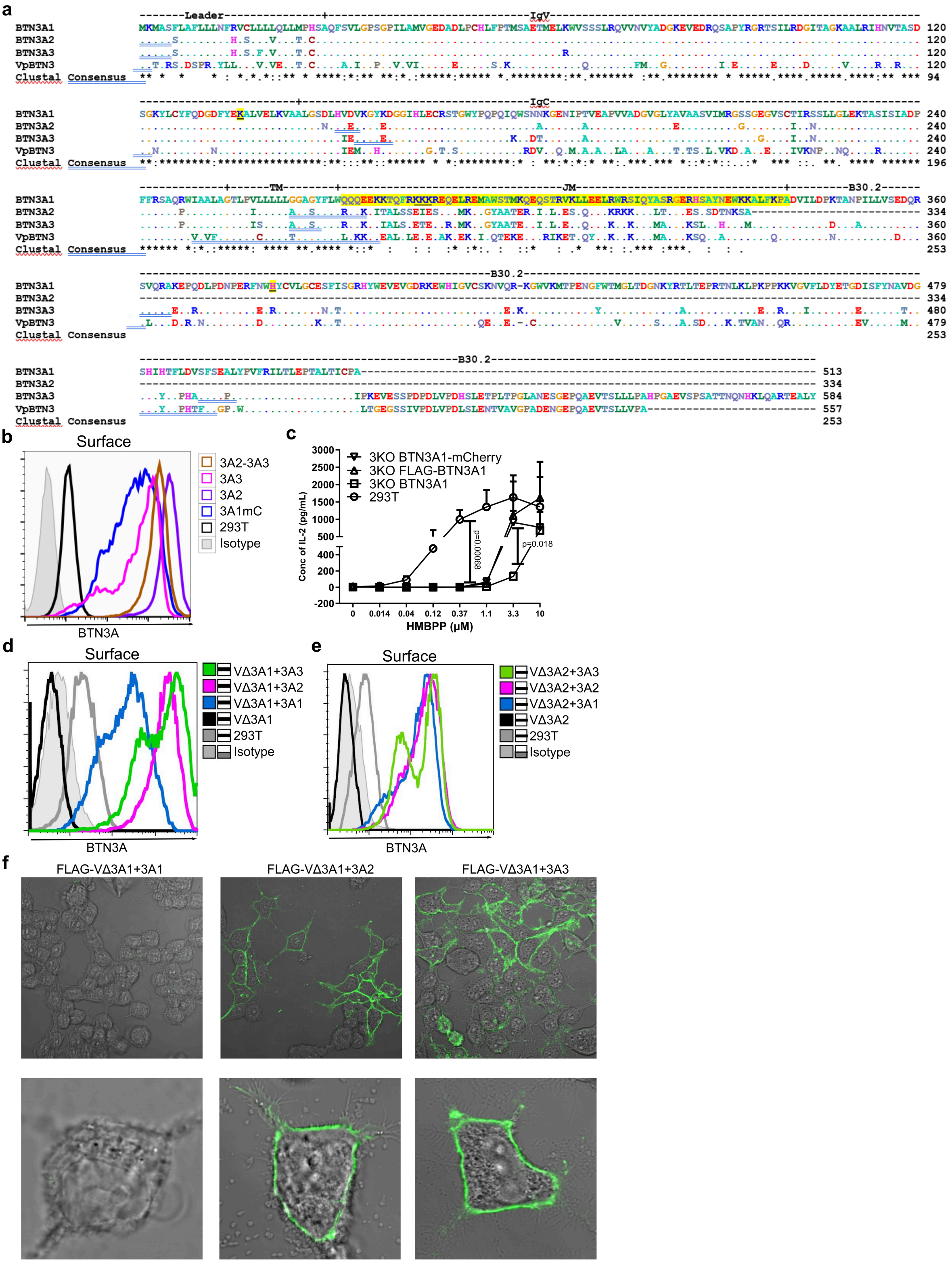

Supplementary Fig 1: Detection of FLAG-VΔ3A at the cell surface in complexes with heteromeric BTN3A.

a The clustal alignment of human BTN3A1, BTN3A2, BTN3A3, and alpaca BTN3 (VpBTN3) protein sequences with conserved amino acids were presented by dots (.). JM is highlighted in yellow and amino acids of interest and their positions are as follows. Highlighted in yellow and underlined: K136, H381, JM region 'KKK' motif of BTN3A1. The clustal consensus revealed the conserved amino acids with asterisks for identical conservation and dots of similar amino acid conservation. **b** The surface-expressed 3A1mC, 3A2, 3A3, or 3A2+3A3 of 3KO transductants detected by mAb 103.2 were presented as histograms. **c** 3KO cells transduced with 3A1 or N-terminus FLAG-tagged 3A1 (FLAG-3A1) or C-terminus mCherry fused 3A1 (3A1mC) in parallel with 293T were treated with titrated concentrations of HMBPP and cocultured with 53/4 human Vγ9Vδ2 TCR reporter cells. The activation of reporter cells was measured via mouse IL-2 ELISA (n-3). Graphical data are presented as mean with SD were analyzed by ordinary two-way ANOVA and SD was shown as error bars. **d** Surface-expressed BTN3A of 293T, 3KO transduced with FLAG-VΔ3A1 alone or with FLAG-VΔ3A1 and 3A-molecules detected by mAb 103.2 followed by anti-mouse F(ab')2-APC conjugate (right) were presented as histograms. **e** The same for 293T, 3KO transduced with FLAG-VΔ3A2 alone or with FLAG-VΔ3A2 and 3A-molecules (right). **f** 3KO cells cotransduced with FLAG VΔ3A1 and 3A1 (left) or 3A2 (center) or 3A3 (right) were stained with mouse-anti-FLAG followed by anti-mouse Alexa Fluor 488 under live conditions. The images captured with confocal microscopy were presented.

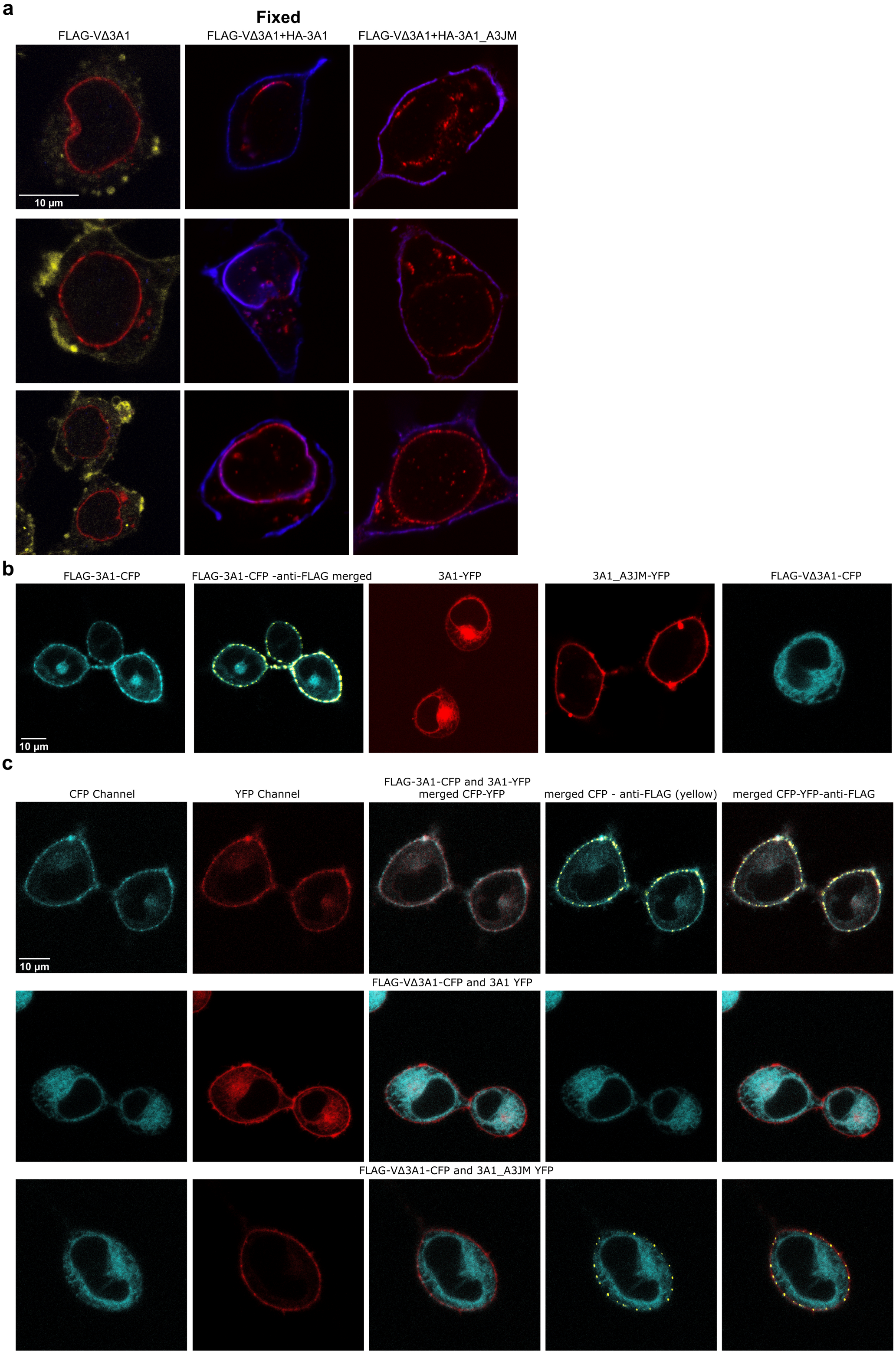
a
Fixed
FLAG-VΔ3A1
FLAG-VΔ3A1+HA-3A1
FLAG-VΔ3A1+HA-3A1_A3JM
10 µm
b
FLAG-3A1-CFP
FLAG-3A1-CFP -anti-FLAG merged
3A1-YFP
3A1_A3JM-YFP
FLAG-VΔ3A1-CFP
10 µm
c
CFP Channel
YFP Channel
FLAG-3A1-CFP and 3A1-YFP
merged CFP-YFP
merged CFP - anti-FLAG (yellow)
merged CFP-YFP-anti-FLAG
10 µm
FLAG-VΔ3A1-CFP and 3A1 YFP
FLAG-VΔ3A1-CFP and 3A1_A3JM YFP

d

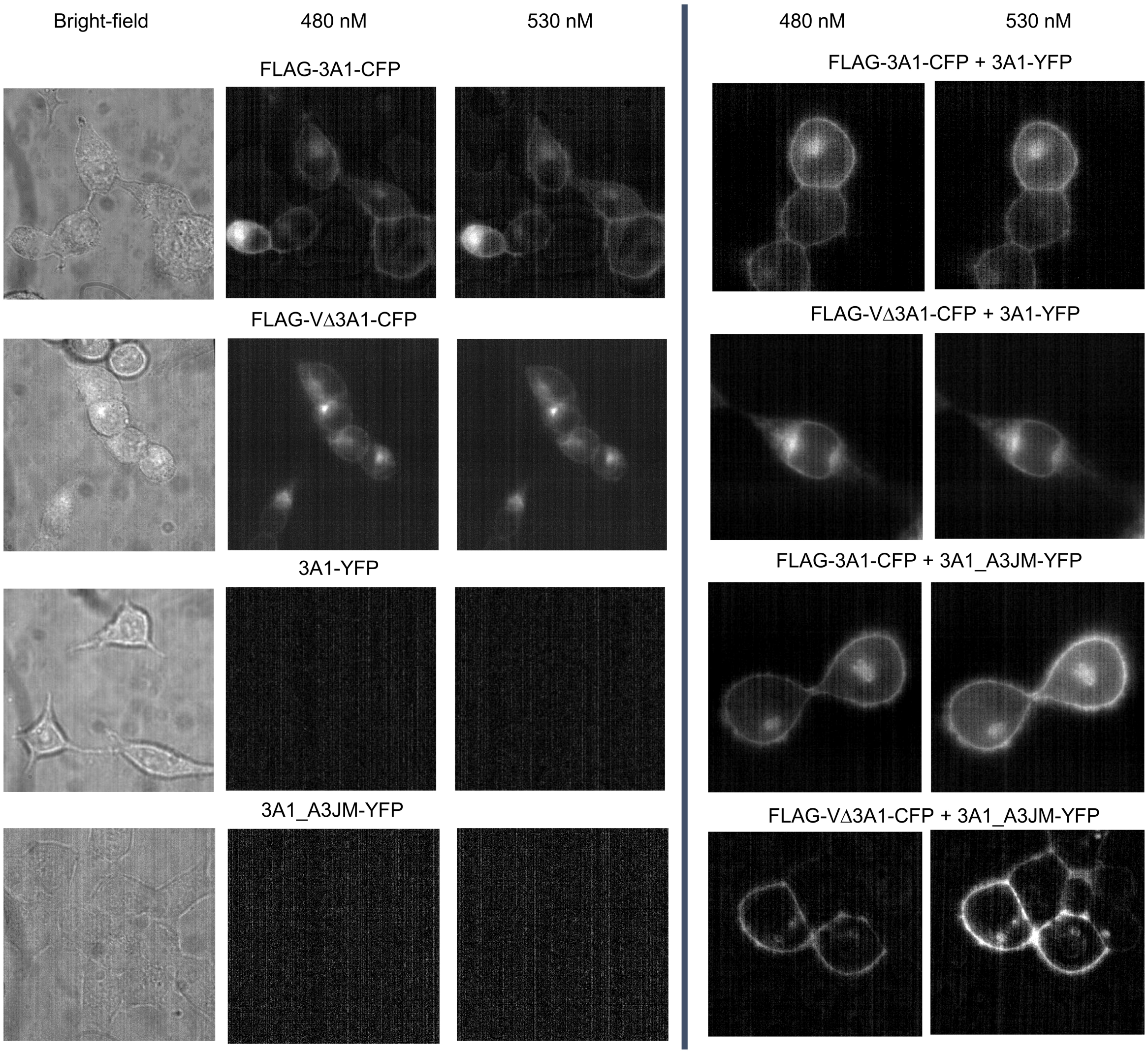

e

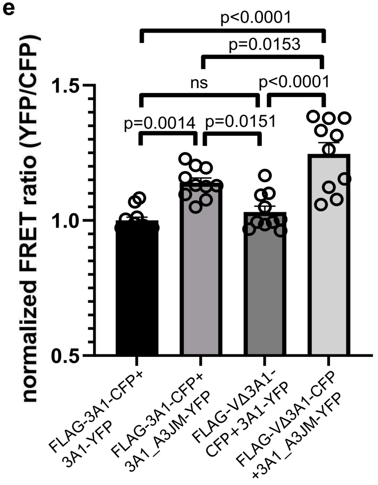

Supplementary Fig 2: Confocal imaging and FRET with live cells demonstrating BTN3A3_JM modulating BTN3A complexes.

a The confocal microscopy images of fixed 3KO cells transduced with FLAG-VΔ3A1, FLAG-VΔ3A1 + HA-3A1 or FLAG-VΔ3A1 + HA-3A1_A3JM chimera were stained with mouse anti-FLAG and rabbit anti-HA followed by anti-mouse-Alexa Fluor 647 (red) and anti-rabbit Alexa Fluor 555 (blue), respectively. 3KO

FLAG VΔ3A1 cells were also stained with BODIPY-FL-DHPE as membrane dye (yellow) to distinguish membrane and perinuclear space staining for FLAG VΔ3A1. Confocal microscopy images by 458, 514, and 648 lasers for the detection of CFP (cyan), YFP (red), and FLAG (mouse anti-FLAG+anti-mouse AF647, represented in yellow) respectively, of **b** Live 3KO cells transduced with FLAG 3A1-CFP (CFP channel) and with anti-FLAG stained (cyan and yellow); 3A1-YFP (YFP channel); 3A1_A3JM-YFP (YFP channel), FLAG VΔ3A1-CFP (CFP channel). **c** Live 3KO cells co-transduced with FLAG 3A1-CFP and 3A1-YFP (upper lane); 3KO cells co-transduced with FLAG VΔ3A1-CFP and 3A1-YFP (middle lane); 3KO cells co-transduced with FLAG VΔ3A1-CFP and 3A1_A3JM-YFP (lower lane) were analyzed as in *A* for the detection of CFP (cyan), YFP (red) and FLAG (mouse anti-FLAG+anti-mouse AF647, represented in yellow) respectively, and for colocalization of CFP, YFP (red) and FLAG (yellow). **d** Bright-field images, phase contrast images detected by 480 and 535 nM filters of 3KO cells transduced with either FLAG-3A1-CFP, FLAG-VΔ3A1-CFP, 3A1-YFP or 3A1_A3JM-YFP (left) and cotransductants of FLAG-3A1-CFP/FLAG-VΔ3A1-CFP with 3A1-YFP or 3A1_A3JM-YFP. **e** The quantitative analysis of total FRET (cytoplasmic and membrane fluorescence), presented as normalized FRET ratio calculated from 10 cells/group (n-10), and ordinary one-way ANOVA was used for statistical analysisand mean values with the SD were presented in graphs.

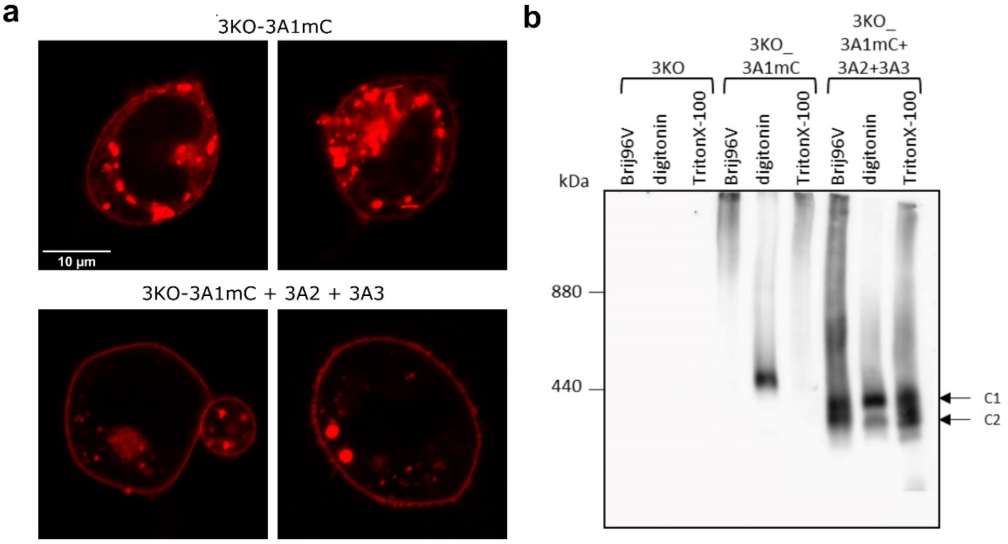

Supplementary Fig 3: Optimal PAg response of 3A1 requires cooperation of 3A2 and 3A3.

a The cellular distribution of 3A1mC fusion proteins expressed in 3KO (upper lane) or 3KO transduced with 3A2+3A3 (bottom lane) are presented as images captured by confocal microscopy. **b** 3KO cells, 3KO expressing 3A1mC (3KO_3A1mC) or 3A1mC+3A2+3A3 were lysed with different detergents, and the lysates were analyzed on a blue native PAGE as follows and C1, C2 represents two complexes with different molecular mass, observed on the blot. Membrane fractions were prepared by disrupting $4x10^7$ cells in 1 ml of a hypotonic buffer (10 mM Hepes, pH7.4, 42 mM KCl, 5 mM $MgCl_2$, and protease inhibitors) using a homogenizer and pelleting the membranes in an ultracentrifuge at 120,000 g. Membranes were lysed in BN lysis buffer (20 mM Bis-Tris, 500 mM ε-aminocaproic acid, 20 mM NaCl, 2 mM EDTA, pH 8.0, 10% glycerol and protease inhibitors) including the detergent indicated. An equivalent of $1x10^7$ cells per 10 µl of BN lysis buffer was used. Samples were separated by BN-PAGE (4–10%) at 4°C as referenced in Materials and Methods. After the gel separation proteins were detected by Western Blotting using an anti-CD277 antibody (mAb 20.1; Invitrogen) and an HRPO-coupled anti-mouse antibody.

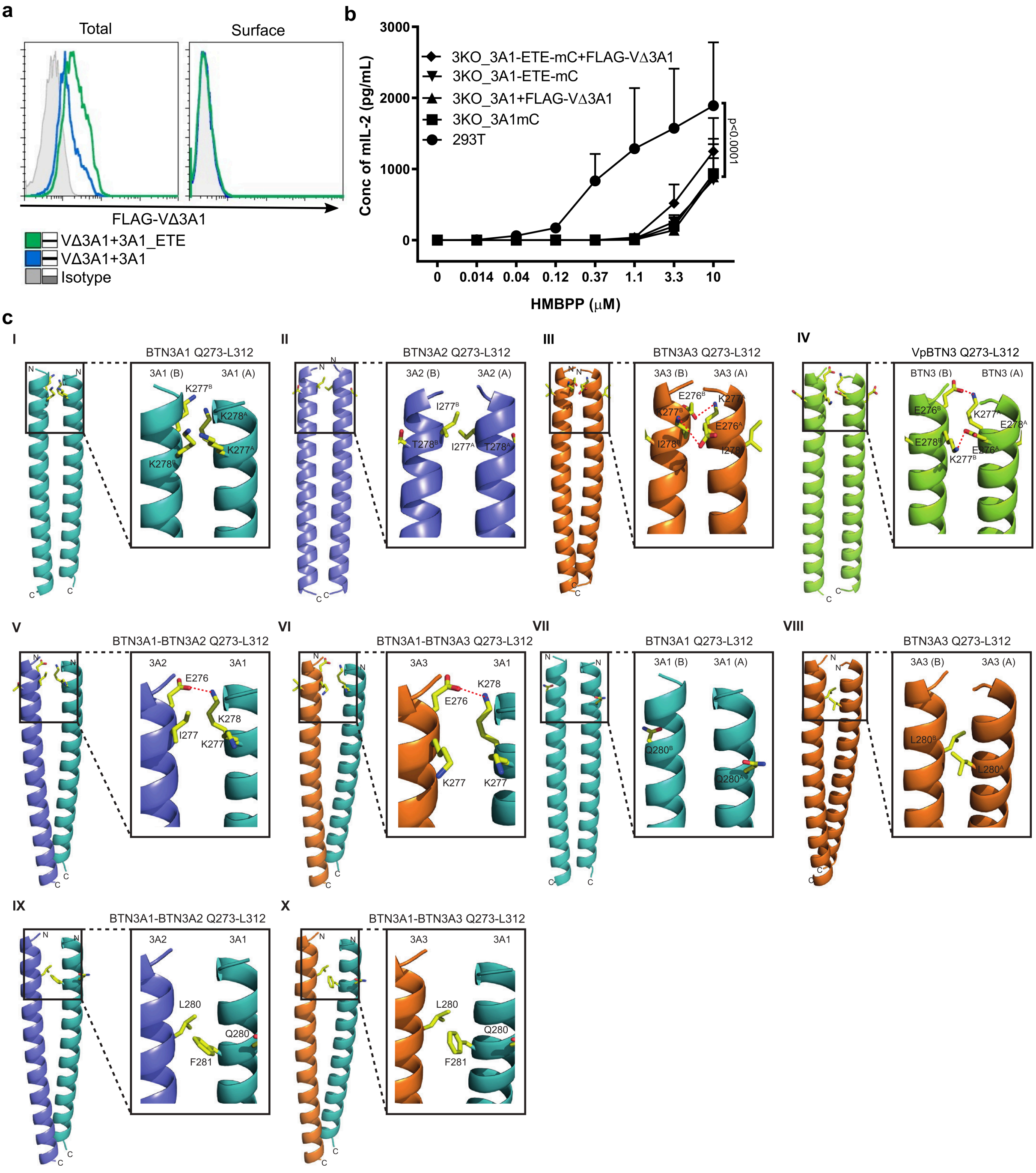

Supplementary Fig 4: Critical residues in JM controlling the association in BTN3A complexes.

a 3KO cells transduced with FLAG-VΔ3A1 alone or cotransduced with 3A1 or 3A1_ETE mutant were analyzed for total and surface-expressed FLAG detected by anti-FLAG, followed by anti-mouse F(ab')2-APC conjugate. The measurements were presented as histograms. **b** 293T and 3KO cells transduced with FLAG-VΔ3A1 and 3A1 or 3A1_ETE mutant were cocultured with titrated concentrations of HMBPP and 53/4 human Vγ9Vδ2 TCR reporter cells. The activation of reporter cells was measured by mouse IL-2 ELISA (left) (n-3). Graphical data are presented as mean with SD were analyzed by ordinary two-way ANOVA and SD was shown as error bars. **c** Models of the predicted JM coiled-coil dimers Q273-

L312 were generated using CCBuilder2 (see Methods). Dimer interface residues are shown as ball and stick. I) BTN3A1 coiled-coil homodimer (focused on residues 276-278). II) BTN3A2 coiled-coil homodimer (focused on residues 276-278). III) BTN3A3 coiled-coil homodimer (focused on residues 276-278). IV) Alpaca BTN3 (VpBTN) coiled-coil homodimer (focused on residues 276-278). V) BTN3A1-BTN3A2 coiled-coil heterodimer (focused on residues 276-278). VI) BTN3A1-BTN3A3 coiled-coil heterodimer (focused on residues 276-278). VII) BTN3A1 coiled-coil homodimer (focused on residue 280). VIII) BTN3A3 coiled-coil homodimer (focused on residue 280). IX) BTN3A1-BTN3A2 coiled-coil heterodimer (focused on residue 280). X) BTN3A1-BTN3A2 coiled-coil heterodimer (focused on residue 280). Polar interactions are highlighted (red dashed lines). Each monomer within the homodimer has been labeled A or B.

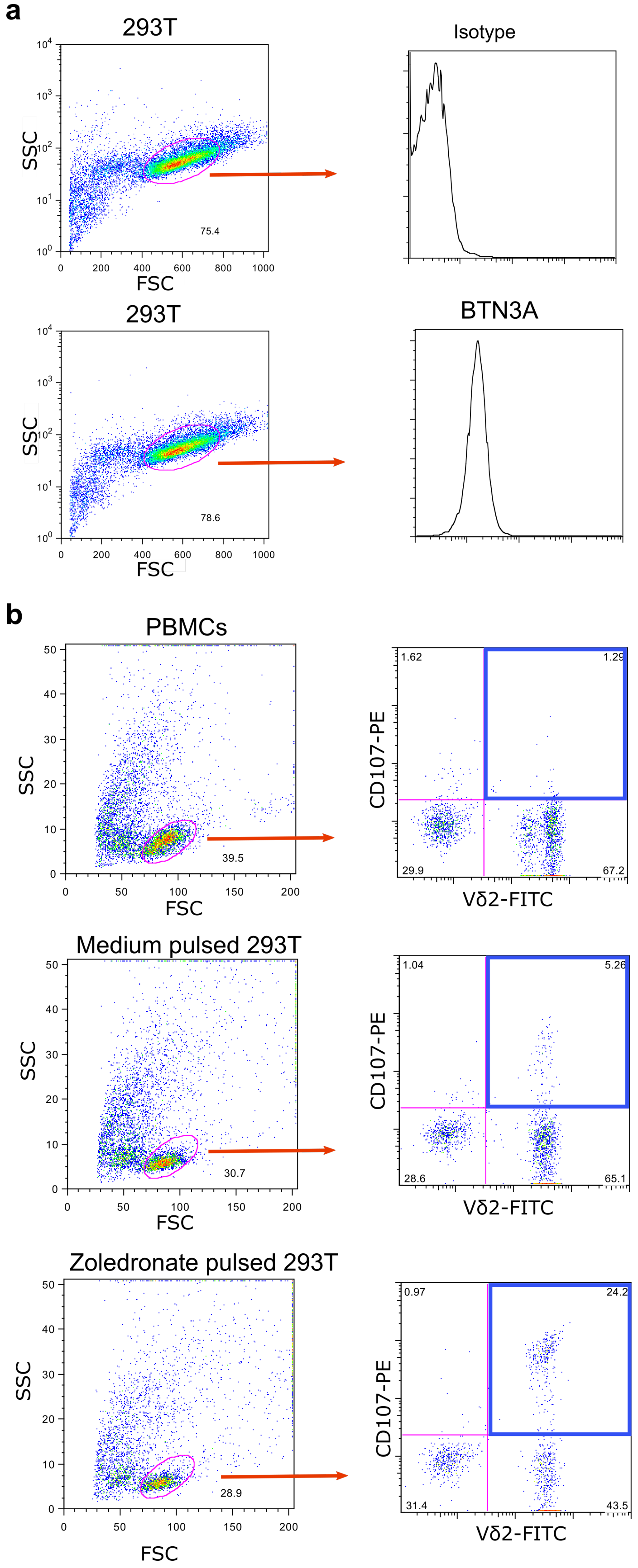

Supplementary Fig 5: Gating strategy for FACS analysis.

a Representative for the analysis of 293T cells for their surface expression, where the population of interest (oval pink) was defined by FSC and SSC. The expression of the protein of interest was shown as histograms (isotype or BTN3A). Long ticks in X-axis represent the logarithmic scale. **b** Representative for the analysis of PBMC alone or cocultured with 293T. The lymphocyte (oval pink) population was defined by FSC and SSC and it was subjected to Vδ2-FITC vs CD107a-PE gating. The population size of each quadrant was shown in numbers and, Vδ2-FITC $^{+}$ and CD107a-PE $^{+}$ (blue quadrant) were analyzed and presented as graphs.

Supplementary Text:

Extra-triplet effects contribute to differential dimer stability.

Based on a parametric α-helical coiled-coil prediction methodology (CCBuilder 2.0), two regions outside residues 283-285 emerged as likely to differentially affect dimer stability. In particular, K277 and K278 in BTN3A1 are predicted to be closely opposed at the interhelical interface, again contributing to a destabilized BTN3A1 homodimer (Figure S4C I). In contrast, in BTN3A2, I277 is predicted to form favorable non-polar interactions with the identical residue across the coiled-coil interface (Figure S4C II). In BTN3A3 and alpaca BTN3, K277 is predicted to form a salt bridge with the preceding E276 from opposing monomer, with a reciprocal of E276 with K277 from the other chain (Figures S4C III-IV). We predict this arrangement, in combination with changes at 283-285, may considerably stabilize the BTN3A3 and alpaca BTN3 relative to BTN3A1 homodimers. Interactions mediated by this region are also likely to preferentially favor BTN3A1/A2 and BTN3A1/A3 heterodimers vs BTN3A1 homodimer formation (Figures S4C V-VI). Of note, the BTN3A1/3A2 and BTN3A1/A3 dimers suggest elimination of electrostatic repulsive effects mediated by opposition of K277 and K278 across the dimer interface, and suggests the heterodimer is likely to benefit from K278 of BTN3A1 interaction with E276 of 3A2/3A3 (Figure S4C V-VI), an interaction not predicted by BTN3A1 homodimer model. In addition, the presence of Q280 in BTN3A1 is also likely significant (Figure S4C VII), as in all other BTN3 isoforms (homodimers/heterodimers), L280 stabilizes the dimer interface via non-polar packing interactions (Figures S4C VIII-X), whereas in BTN3A1 these are absent, with Q280 remaining polar and solvent exposed. This difference also likely favors BTN3A1 heterodimer formation, as L280 in BTN3A2 and BTN3A3 can also mediate non-polar stacking interactions with F281 (in BTN3A1) (Figures S4C IX-X); in contrast, in BTN3A1 Q280 remains solvent exposed (Figure S4C VII). Notably, the single Alpaca isoform BTN3 retains both L280 and F281 at the interhelical interface, contributing to coiled-coil stabilization. Thus, clearly interhelical interactions outside of the 283-285 region also preferentially destabilize BTN3A1 homodimers relative to both BTN3A2/3 homodimers, and also relative to BTN3A1 heterodimers with BTN3A2/A3. This provides molecular justification for

the finding that introduction of the 283-285 ETE sequence of BTN3A3 into BTN3A1 is insufficient to substantially increase expression and functionality.

Synthesis of 4-hydroxy-3-(4-methylbenzyl)but-2-en-1-yl diphosphate (4-M-HMBPP)

To a stirred suspension of NaH (60 % in oil, 26.25 mmol) in THF (50 ml) was added ethyl (diethoxyphosphoryl) acetate (**2**) (25 mmol) at 0 °C. The mixture was stirred for 1 hour and *p*-methyl benzyl bromide (**1**) (30 mmol) was then added. The mixture was stirred at room temperature overnight and then quenched with saturated NH_4Cl at 0 °C. The aqueous layer was extracted with Et_2O, dried over $MgSO_4$ and evaporated to give a yellow oil which was purified by silica gel column chromatography to give (**3**) with the yield of **71%**. In the next step, to a stirred suspension of NaH (60% in oil, 2.2 mmol) in THF (5 ml) was added a solution of (**3**) (2 mmol) in THF (4 mL) at 0 °C. Then, the mixture was allowed to stir at room temperature for 1 h and cooled to 0 °C, again. Then a solution of chloroacetaldehyde (50 wt% in H_2O) in THF (1 ml) was added. The reaction mixture was then stirred at room temperature for 2 hours, diluted with ether and washed with brine. The organic layer was dried over $MgSO_4$ and evaporated to give a yellow oil, which was further purified by silica gel column chromatography to obtain *E:Z* mixture of (**4**) with the yield of **68%**. Later, the same mixture of compound (**4**) (2 mmol) was dissolved in CH_2Cl_2 (10 mL) and then slowly added DIBAL (1.2 M 20 wt% solution in toulene, 4.2 mL) at -78 °C under argon. When the starting material was consumed, as monitored by TLC, the reaction was quenched with aqueous sodium potassium tartrate. The aqueous layer was extracted with CH_2Cl_2 and the combined organic layers dried with $NaSO_4$, and concentrated. The yellow residue was purified by flash chromatography on silica gel to afford the desired product (**5**) again as *E:Z* mixture with yield of **57%**. In the last step tris(tetra-*n*-butylammonium) hydrogen diphosphate (4 mmol) was dissolved in MeCN and *E:Z* mixture of (**5**) (2 mmol) was added and stirred for 2 h., purified by Dowex (NH_4^+) followed by Sephadex LH-20 using methanol as solvent to yield the final product (**6**), **21%**.

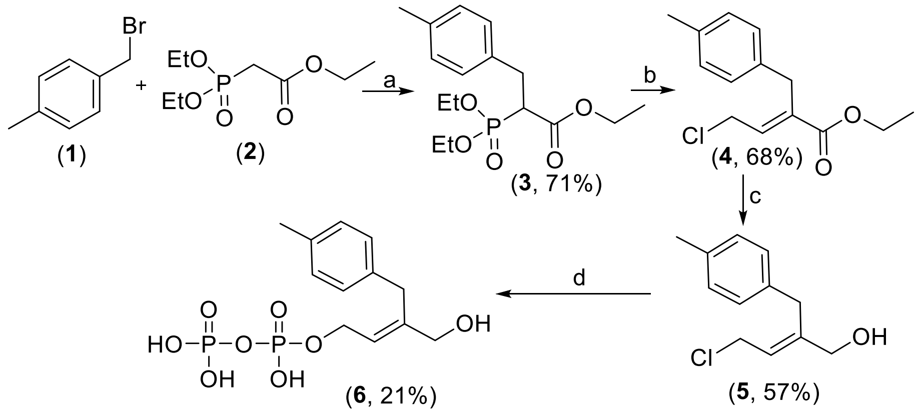

Reagents and conditions: (a) NaH/THF, 0 °C; (b) NaH/THF, chloroacetaldehyde (50%, aq); (c) DIBAL, -78 °C, Argon; (d) tris(tetra-n-butylammonium) hydrogen diphosphate, MeCN, Dowex (NH_4^+), Sephadex (LH-20)

Spectral Data:

ethyl 2-(diethoxyphosphoryl)-3-(p-tolyl)propanoate (3): Yellow oil, 71% yield, ^{1}H NMR ($CDCl_3$, 500 MHz) δ ppm 7.10(s, 4H), 4.24-4.14(m, 5H), 4.13-4.11(m, 2H), 3.28-3.17(m, 2H), 2.33(s, 3H), 1.39-1.36(m, 6H), 1.20-1.17(m, 3H)

ethyl (*E*)-4-chloro-2-(4-methylbenzyl)but-2-enoate (4): Yellow oil, 68% yield, ^{1}H NMR ($CDCl_3$, 500 MHz) (*E:Z* mix 4:1) δ ppm 7.15-7.11(s, 4H), 7.02-6.99(t, *J* = 10, 1H), 4.29-4.28(d, *J* = 5, 2H), 4.25-4.20(m, 2H), 3.75(s, 2H), 2.37(s, 3H), 1.32-1.29(t, *J* = 10, 3H)

4-chloro-2-(4-methylbenzyl)but-2-en-1-ol (5): Yellow oil, 57% yield ^{1}H NMR ($CDCl_3$, 500 MHz) (*E:Z* mix 3:1) δ ppm 7.19-7.13(s, 5.55H), 5.95-5.92(m, 1H), 5.69-5,66(m, 0.37H), 4.30-4.28(m, 2H), 4.24-4.22(m, 0.78H), 4.20(s, 0.77H)4.05(s, 2H), 3.53(s, 2H)3.52(s, 0.78H)2.39-2.38(m, 4H) 1.90(s, 1.38H)

4-hydroxy-3-(4-methylbenzyl)but-2-en-1-yl diphosphate (6): white semisolid, 21% yield ^{1}H NMR (CD_3OD, 500 MHz) (*E:Z* mix 8.3:1.7) δ ppm 7.12-7.06 (s, 4H), 5.88 (s, 1H), 4.78 (s, 2H), 3.89 (s, 2H), 3.49 (s, 2H), 3.37(s, 2H), 2.30 (s, 3H); ^{31}P NMR(CD_3OD, 202 MHz) δ ppm -9.82(s, 1P), -10.23(s, 1P); ^{13}C NMR (CD_3OD, 125 MHz) δ ppm 141.87, 140.93, 136.29, 136.26, 135.16, 135.07, 128.81,128.61, 128.53, 128.31, 125.35, 122.99, 64.39, 62.22, 32.93, 19.73, 19.32

^1H NMR of Compound(6):

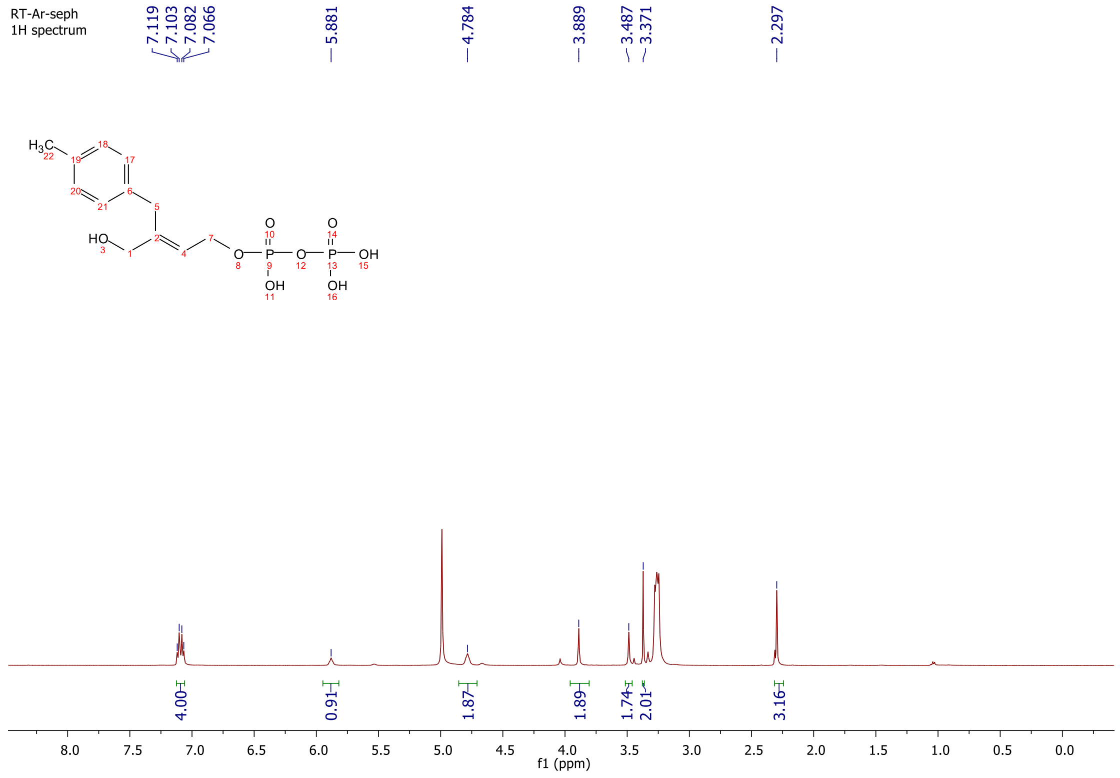

^{13}C NMR of Compound(6):

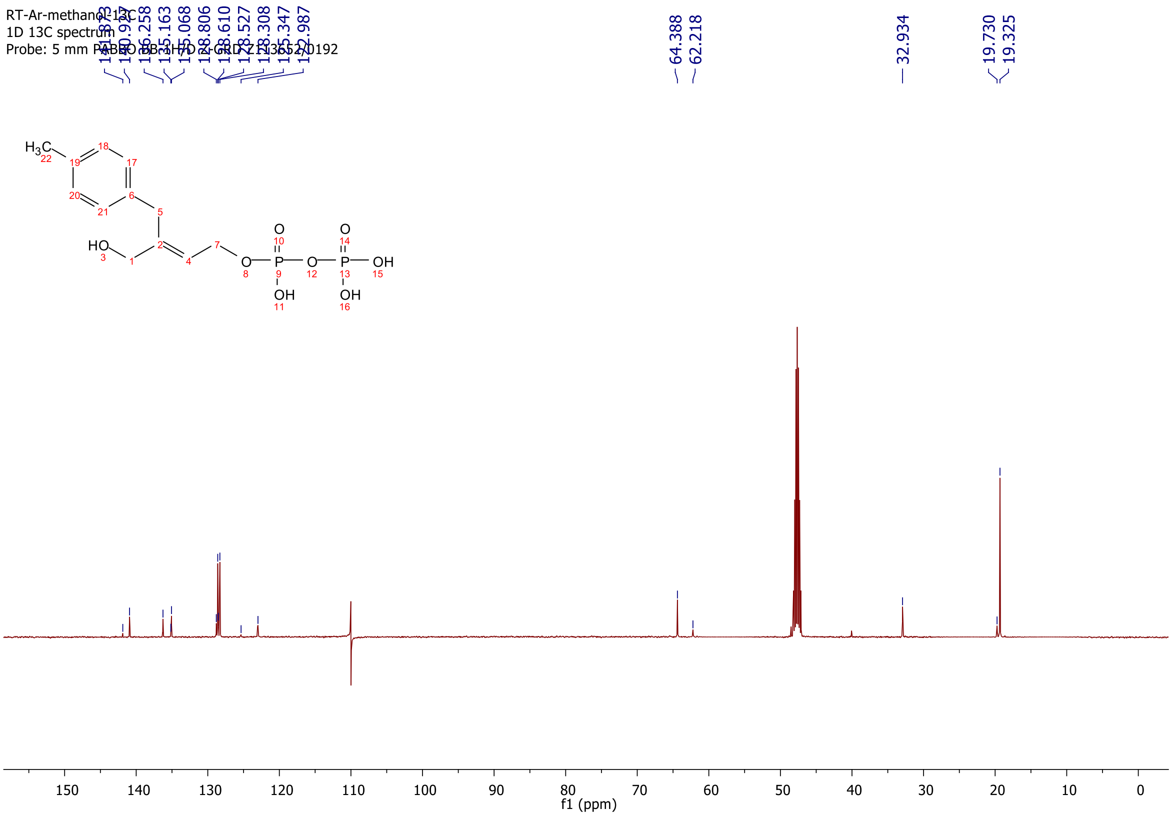

^{31}P NMR of Compound(6):

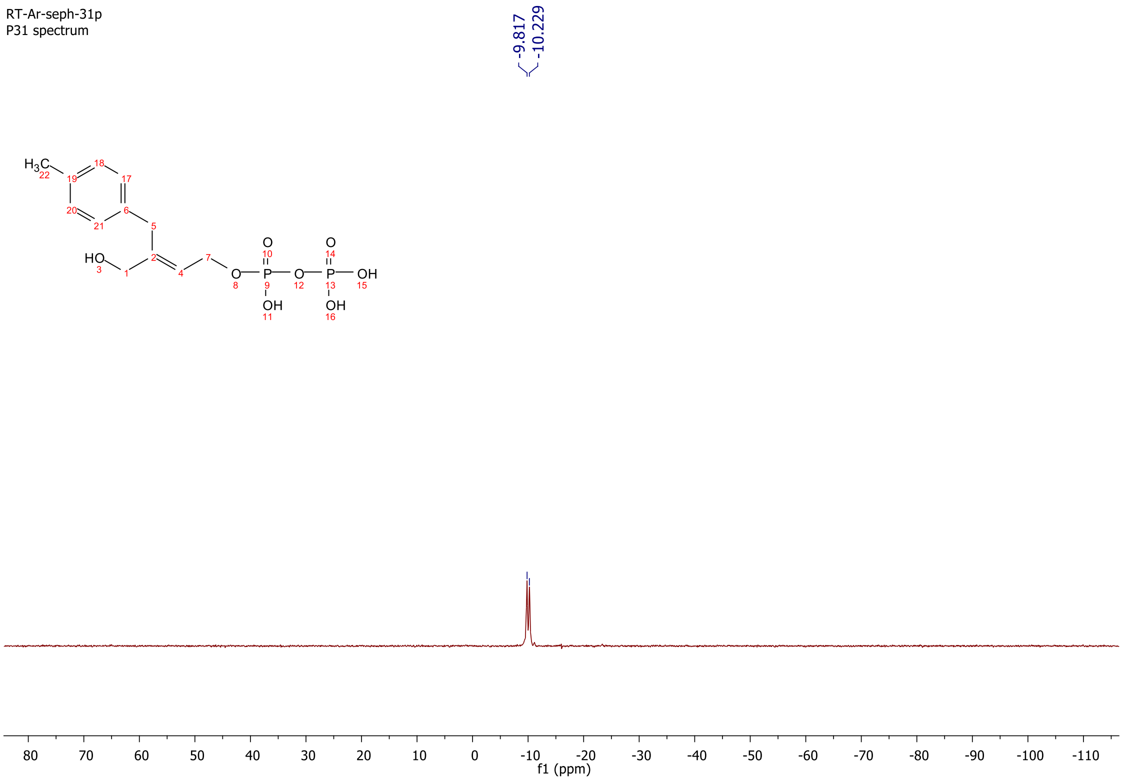

Supplementary Table 1: CRISPR and primer sequences

**Primer Name: Sequence**
**CRISPR sequences**
BTN3CrispR3Fwd: TCCCGAGGGATCCATCTGGAGTGC
BTN3CrispR3Rev: AAACGCACTCCAGATGGATCCCTC
BTN3A1CrispR4Fwd: TCCCGGGAGAGAACATCCCGACTG
BTN3A1CrispR4Rev: AAACCAGTCGGGATGTTCTCTCCC
BTN3A2CrispR6Fwd: TCCCGCATCTGTGATCATGAGAGG (CRISPR Sequence is present in all BTN3 genes but only BTN3A2 possess Sp Cas9 specific PAM sequence (CGG)
BTN3A2CrispR6Rev: AAACCCTCTCATGATCACAGATGC
BTN3A3CrispR7Fwd: TCCCATAAAGTGGAGCGACACCAA
BTN3A3CrispR7Rev: AAACTTGGTGTCGCTCCACTTTAT
**Primer sequences**
BTN3A1GenoIgCFp: GGCCTCTCAAGAATTTAGGCT
BTN3A1GenoIgCRp: AATGTTCAGTAAATGTTTGCGTCA
BTN3A2GenoIgCFp: TCCCTCATCATGACCACACTTTT
BTN3A2GenoIgCRp:ACGAACACCTTTATGGAGGAAAGA
BTN3A3GenoIgCFp: GGATGGGAATAGCCCCACATTAC
BTN3A3GenoIgCRp: CCTAAAGCAGTGAGTAGCCTTCA
BTN3A1EcoR1KozakFwd: CTATGAATTCGCCGCCACCATGAAAATGGCAAGTTTCCTGGC
BTN3A1-BglIIRev: CTACAGATCTTCACGCTGGACAAATAGTCAGGG
BTN3A2_Xba1KozakFwd: CTATTCTAGAGCCGCCACCATGAAAATGGCAAGTTCCCTGG

BTN3A2_T2Arev<br>CCAGGATTCTCCTCGACGTCACCGCATGTTAGCAGACTTCCTCTGCCCTCGGCTGACTTATTGGTATCGGAC
BTN3A3_T2Afwd:<br>GAGGGCAGAGGAAGTCTGCTAACATGCGGTGACGTCGAGGAGAATCCTGGCCCAATGAAAATGGCAAGTTCCCTGG
BTN3A3_EcoR1Rev: CTACGAATTCTCAGTAAAGTGCTTCAGTGCGT
BTN3A2_EcoR1Rev: CTACGAATTCTCAGGCTGACTTATTGGTATCG
BTN3A2_EcoR1KozakFwd: ATGAATTCGCCGCCACCATGAAAATGGCAAGTTCCCTGG
BTN3A2_Swa1Rev: CTACATTTAAATTCAGGCTGACTTATTGGTATCG
BTN3A3_Swa1Rev: CTACATTTAAATTCAGTAAAGTGCTTCAGTGCGT
FLAGtagBTN3A1Fwd: CAAGGCTAGATCTCAGTTTTCTGTGCTTGGAC
FLAGtagBTN3A1IgVDelFwd: CAAGGCTAGATCTCTGGGTTCTGATCTTCACG
BTN3A1EcoRIRev: TATTGAATTCTCACGCTGGACAAATAGTCAG
EcoRI-KOZAK-HA-Hu-3A1Fwd:<br>ggaccatcctctagaGAATTCGCCGCCACCATGAAAATGGCAAGTTTCCTGGCCTTCCTTCTGCTCAACTTTCGTGTCTGCCTCCTTTTGCTTCAGCTGCTCATGCCTCACTCAGCT
HA-Hu3A1LdrRev:<br>CAGAGGGTCCAAGCACAGAAAACTGGGATCCAGCCTTGTCGTCATCGTCTTTGTAGTCTGCTGATCCAGCTGAGTGAGGCATGAGCAGCTGA
BTN3A1IgVfwd: CAGTTTTCTGTGCTTGGACCCTCTG
pIZ-BamHuBTN3A1Rev: GGGGGAGGGAGAGGGGGATCCTCACGCTGGACAAATAGTCAGGG
pHNGFR_3A1-A3JTM inHDFU Fwd: TCCTCTAGAGAATTCGCCGCCACCATGAAAATGGCAAGTTTCCTG
pHNGFR_3A1-A3JTM inHDFU Rev: CTGCTTCTGCGGATCCCGCTGGACAAATAGTCAGGG
pHNGFR_3A3-A1JTM inHDFU Fwd: TCCTCTAGAGAATTCGCCGCCACCATGAAAATGGCAAGTTTCCTG
pHNGFR_3A3-A1JTM inHDFU Rev: CTGCTTCTGCGGATCCGTAAAGTGCTTCAG
K136A_BTN3A1-Fwd: ATGGTGACTTCTATGAAGCAGCCCTGGTGG
K136A _BTN3A1-Rev: CCACCAGGGCTGCTTCATAGAAGTCACCAT
BTN3A1_H381R-Fwd: CCCTGAGAGATTTGAATGGCGTTACTGTGTCCTTGGC
BTN3A1_H381R-Rev: GCCGAGAACACAATAACGCCAATTAAATCTCTCAGGG
BTN3A3_R381H-Fwd: CCCTGAGAGATTTGAATGGCATTACTGTGTCCTTGGC
BTN3A3_R381H-Rev: GCCAAGGACACAGTAATGCCATTCAAATCTCTCAGGG
BTN3A1_ETE_Fwd: GAGACAGAGAGAGAGCAAGAGTTGAGAGAA
BTN3A1_ETE_Rev: CTCTCTCTCTGTCTCTCTGAACTGAGTCTT
BTN3A3_KKK_Fwd: AAGAAAAAAAGAGAGCGAGAGATGAAAGAA
BTN3A3_KKK_Rev: CTCTCTTTTTTTCTTCCTGGACAGAGCAAT
pIH-MCS: TGGACCATCCTCTAGAgaattcAATATTTAAATACCATGGCAATTggatccCCCTCT
pIH-FLAG MCS:<br>gaattgCCACCATGAAA**ATG**GCAAGTTTCCTGGCCTTCCTTCTGCTCAACTTTCGTGTCTGCCTCCTTTTGCTTCAGCTGCTCATGCCTCACTCAGCT<u>GGATCAGCA</u>*GACTACAAGGACGACGATGACAAG*<u>GCTggatcc</u>CACCATGGTAATTgaattcAATATTTAAATAATagatcc

pIZ-MCS: TGGACCATCCTCTAGAgaattcAATATTTAAATACCATGGCAATTggatccCCCTCT
pIZ-HA:<br>gaattcGCCGCCACCATGAAAATGGCAAGTTTCCTGGCCTTCCTTCTGCTCAACTTTCGTGTCTGCCTCCTTTTG<br>CTTCAGCTGCTCATGCCTCACTCAGCTGGATCAGCATACCCATACGATGTTCCAGATTACGCTGCTggatcc

Supplementary Table 2: Plasmids and constructs

**Plasmid - Insert**
pIZ-3A2, -3A3, 3A2-T2A-3A3
pIHFLAG-BTN3A1
pIHFLAG-VΔ3A1 and -VΔ3A2
pIZHA-3A1, -3A1_A3JM
pEGZ-3A3 and -3A1_H381R
phNGFR linker mCherry – 3A1
phNGFR linker mCherry - 3A1_A3JM, -3A3-R381H, -3A3_A1JM-381H
phNGFR linker CFP – 3A1, - FLAG-VΔ3A1
phNGFR linker YFP – 3A1, -3A1_A3JM
FgH1tUTG (CRISPR Sequences)
FuCas9Cherry
pET-21b - BTN3A1-BFI (BFI)
pET-21b – BTN2A1-ID271 (ID271)
