# Supplementary material for: A distinct topology of BTN3A IgV and B30.2 domains controlled by juxtamembrane regions favors optimal human γδ T cell phosphoantigen sensing
Source: Nat Commun. 2023 Nov 22;14:7617. doi: 10.1038/s41467-023-41938-8 (PMC10665462; doi:10.1038/s41467-023-41938-8)
Supplement: Supplementary file 3 — Reporting Sumary [file 41467_2023_41938_MOESM3_ESM.pdf]

nature portfolio

Corresponding author(s): Thomas Herrmann
Mohindar Murugesh Karunakaran
NCOMMS-23-07146

Last updated by author(s): May 25, 2023

Reporting Summary

Nature Portfolio wishes to improve the reproducibility of the work that we publish. This form provides structure for consistency and transparency in reporting. For further information on Nature Portfolio policies, see our Editorial Policies and the Editorial Policy Checklist.

Statistics

For all statistical analyses, confirm that the following items are present in the figure legend, table legend, main text, or Methods section.

n/a	Confirmed	
☐	☒	The exact sample size (*n*) for each experimental group/condition, given as a discrete number and unit of measurement
☐	☒	A statement on whether measurements were taken from distinct samples or whether the same sample was measured repeatedly
☐	☒	The statistical test(s) used AND whether they are one- or two-sided<br>*Only common tests should be described solely by name; describe more complex techniques in the Methods section.*
☒	☐	A description of all covariates tested
☐	☒	A description of any assumptions or corrections, such as tests of normality and adjustment for multiple comparisons
☐	☒	A full description of the statistical parameters including central tendency (e.g. means) or other basic estimates (e.g. regression coefficient) AND variation (e.g. standard deviation) or associated estimates of uncertainty (e.g. confidence intervals)
☐	☒	For null hypothesis testing, the test statistic (e.g. *F*, *t*, *r*) with confidence intervals, effect sizes, degrees of freedom and *P* value noted<br>*Give P values as exact values whenever suitable.*
☒	☐	For Bayesian analysis, information on the choice of priors and Markov chain Monte Carlo settings
☒	☐	For hierarchical and complex designs, identification of the appropriate level for tests and full reporting of outcomes
☒	☐	Estimates of effect sizes (e.g. Cohen's *d*, Pearson's *r*), indicating how they were calculated

Our web collection on statistics for biologists contains articles on many of the points above.

Software and code

Policy information about availability of computer code

Data collection	Flow cytometry by Cell Quest with FACScalibur, Cell sorting by FACS Diva 8 with FACS Aria III, Image Studio of LI-COR OdysseyXF Imager, ZEN of Zeiss LSM 780
Data analysis	FlowJo 10.7.1 for FACS, GraphPad Prism 9 for statistics; Image Studio Lite 5.2 for western blotting, and Image J (1.53) for imaging data analysis

For manuscripts utilizing custom algorithms or software that are central to the research but not yet described in published literature, software must be made available to editors and reviewers. We strongly encourage code deposition in a community repository (e.g. GitHub). See the Nature Portfolio guidelines for submitting code & software for further information.

Data

Policy information about availability of data

All manuscripts must include a data availability statement. This statement should provide the following information, where applicable:

- Accession codes, unique identifiers, or web links for publicly available datasets
- A description of any restrictions on data availability
- For clinical datasets or third party data, please ensure that the statement adheres to our policy

N/A

Research involving human participants, their data, or biological material

Policy information about studies with human participants or human data. See also policy information about sex, gender (identity/presentation), and sexual orientation and race, ethnicity and racism.

Reporting on sex and gender	N/A see below
Reporting on race, ethnicity, or other socially relevant groupings	N/A no
Population characteristics	N/A Lab members (maler and female) anonymized
Recruitment	N/A Lab members (maler and female) anonymized
Ethics oversight	Enrolled healthy volunteers with informed consent according to the University of Wuerzburg institutional review board (Gz. 20220927 01)

Note that full information on the approval of the study protocol must also be provided in the manuscript.

Field-specific reporting

Please select the one below that is the best fit for your research. If you are not sure, read the appropriate sections before making your selection.

☒ Life sciences ☐ Behavioural & social sciences ☐ Ecological, evolutionary & environmental sciences

For a reference copy of the document with all sections, see nature.com/documents/nr-reporting-summary-flat.pdf

Life sciences study design

All studies must disclose on these points even when the disclosure is negative.

Sample size	Sample sizes were not predetermined and it was based on the availability. For confocal imaging, FRAP and FRET n=>10 samples were analysed for each cell type.
Data exclusions	No data set was excluded
Replication	All the experiments were repeated thrice independently
Randomization	N/A
Blinding	The person performed the experiments were aware of the sample during every experiment therefore blinding was not possible

Reporting for specific materials, systems and methods

We require information from authors about some types of materials, experimental systems and methods used in many studies. Here, indicate whether each material, system or method listed is relevant to your study. If you are not sure if a list item applies to your research, read the appropriate section before selecting a response.

Materials & experimental systems

n/a	Involved in the study	
☐	☒	Antibodies
☐	☒	Eukaryotic cell lines
☒	☐	Palaeontology and archaeology
☒	☐	Animals and other organisms
☒	☐	Clinical data
☒	☐	Dual use research of concern
☒	☐	Plants

Methods

n/a	Involved in the study	
☐	☐	ChIP-seq
☐	☒	Flow cytometry
☐	☐	MRI-based neuroimaging

Antibodies

Antibodies used	Anti-huBTN3 (CD277) clone 103.2 Gift from Daniel Olive<br>Anti-huBTN3 (CD277) clone 20.1 Invitrogen 14-2779-82<br>Anti-CD107a-PE (CloneH4A3) BD Pharmingen 555801

	Anti-Vδ2-FITC (Clone IMMU 389) Beckman Coulter IM1464<br>Anti-FLAG (M2) SIGMA F3165<br>Anti-HA (C29F4) CST 3724<br>Anti-HA (F-7) Santa Cruz sc-7392<br>Anti-Vinculin (Clone V284) SIGMA SAB4200080<br>mIgG1 k isotype clone p3.6.2.81 eBiosciences REF:16-4714-85<br>mIgG2a k isotype clone-eBM2a eBiosciences REF:16-4724-85<br>Goat anti-mouse IgG (H+L) – Alexa Fluor TM 488 Invitrogen A11029<br>Goat anti-mouse IgG1 – Alexa Fluor TM 647 Invitrogen A21240<br>Goat anti-rabbit IgG (H+L) - Alexa Flour TM 555 Invitrogen A21248<br>Fab Donkey anti mouse IgG (H+L)-APC Jackson Immunoresearch Code: 115-136-146<br>BODIPY-FL-DHPE ((N-(4,4-Difluoro-5,7-Dimethyl-4-Bora-3a,4a-Diaza-s-Indacene-3-Propionyl)-1,2-Dihexadecanoyl-sn-Glycero-3-Phosphoethanolamine, Triethylammonium Salt) Invitrogen D-3800
Validation	All the antibodies were validated by the manufacturer for Flow cytometry and for western blotting. mAb 103.2 (Clone 103.2) against human BTN3As was well documented (Harly et al 2012, Palakodeti et al 2012) and commercially available at Creativebiolabs (https://www.creativebiolabs.net/Anti-BTN3A1-Recombinant-Antibody-clone-103-2-24595.htm)

Eukaryotic cell lines

Policy information about cell lines and Sex and Gender in Research

Cell line source(s)	293T DSMZ ACC 635 (This was the parent line used to generate 293T BTN3 deficient and BTN3 reconstituted lines), 53/4 hybridoma Vγ9Vδ2 - MOP TCR (Starick et al., 2017)
Authentication	Cells were not authenticated
Mycoplasma contamination	The cell lines are negative for mycoplasm contamination and were tested by PCR (Uphoff, C.C. and Drexler, H.G. (2014), Detection of Mycoplasma Contamination in Cell Cultures. Current Protocols in Molecular Biology, 106: 28.4.1-28.4.14. https://doi.org/10.1002/0471142727.mb2804s106)
Commonly misidentified lines<br>(See ICLAC register)	N/A

Plants

Seed stocks	N/A
Novel plant genotypes	N/A
Authentication	N/A

ChIP-seq

Data deposition

☐ Confirm that both raw and final processed data have been deposited in a public database such as GEO.

☐ Confirm that you have deposited or provided access to graph files (e.g. BED files) for the called peaks.

Data access links<br>*May remain private before publication.*	*For "Initial submission" or "Revised version" documents, provide reviewer access links. For your "Final submission" document, provide a link to the deposited data.*
Files in database submission	*Provide a list of all files available in the database submission.*
Genome browser session<br>(e.g. UCSC)	*Provide a link to an anonymized genome browser session for "Initial submission" and "Revised version" documents only, to enable peer review. Write "no longer applicable" for "Final submission" documents.*

Methodology

Replicates	*Describe the experimental replicates, specifying number, type and replicate agreement.*
Sequencing depth	*Describe the sequencing depth for each experiment, providing the total number of reads, uniquely mapped reads, length of reads and whether they were paired- or single-end.*
Antibodies	*Describe the antibodies used for the ChIP-seq experiments; as applicable, provide supplier name, catalog number, clone name, and lot number.*
Peak calling parameters	*Specify the command line program and parameters used for read mapping and peak calling, including the ChIP, control and index files used.*

Data quality | *Describe the methods used to ensure data quality in full detail, including how many peaks are at FDR 5% and above 5-fold enrichment.*

Software | *Describe the software used to collect and analyze the ChIP-seq data. For custom code that has been deposited into a community repository, provide accession details.*

Flow Cytometry

Plots

Confirm that:

☒ The axis labels state the marker and fluorochrome used (e.g. CD4-FITC).

☒ The axis scales are clearly visible. Include numbers along axes only for bottom left plot of group (a 'group' is an analysis of identical markers).

☒ All plots are contour plots with outliers or pseudocolor plots.

☒ A numerical value for number of cells or percentage (with statistics) is provided.

Methodology

Sample preparation	For the CD107a assay, PBMCs expanded Vγ9Vδ2 T cells were cocultured with 293T cells along with anti-CD107a-PE (BD Pharmingen) conjugated antibody and cultured for 4 hours. After 4 hours, the cells were collected from the wells as triplicates and washed once with PBS. After which cells were treated with anti-human Vδ2-FITC (Beckman Coulter) conjugated antibody for 20 mins and washed once, followed by analysis at FACSCalibur (BD) for the percentage of Vδ2-FITC and CD107a-PE population. For analysis of total or surface expression of BTN3 in 293T cells, cells were fixed with fixation buffer for 30 mins at RT, followed by wash and incubated for 30 mins with permeabilization buffer at RT. Then cells were stained with antibodies that were prediluted in permeabilization for 30 mins at 4°C, as per the manufacturer's instructions (eBiosciences, eBiosciencesTM Intracellular Fixation & Permeabilization buffer set). For surface staining, cells were directly stained with antibodies of interest for 30 minutes mins at 4°C. The BTN3As were detected by unconjugated mAb 103.2 (gift from Daniel Olive). If tagged, unconjugated anti-FLAG (M2, SIGMA) and anti-HA (F-7, Santa Cruz) antibodies were used. The primary antibodies were detected by Fab Donkey anti mouse IgG (H+L)-APC (Jackson Immunoresearch, 115-136-146). mIgG1k and mIgG2a k (eBiosciences) were used as isotype controls.
Instrument	FACSCalibur (BD)
Software	Cell Quest and FlowJo 10.7.1
Cell population abundance	N/A
Gating strategy	The cells were primarily gated for FSC and SSC to define the gate of interest. That was further analyzed based on the antibody and flurophore conjugates that differentiated the positive and negative population. For all staining isotype and negative controls controls were used as controls. Most FACS analysis represented the expression of BTN3A molecules, and they were presented as histograms in which the long ticks in X-axis represent the logarithmic scale

☒ Tick this box to confirm that a figure exemplifying the gating strategy is provided in the Supplementary Information.

Magnetic resonance imaging

Experimental design

Design type	*Indicate task or resting state; event-related or block design.*
Design specifications	*Specify the number of blocks, trials or experimental units per session and/or subject, and specify the length of each trial or block (if trials are blocked) and interval between trials.*
Behavioral performance measures	*State number and/or type of variables recorded (e.g. correct button press, response time) and what statistics were used to establish that the subjects were performing the task as expected (e.g. mean, range, and/or standard deviation across subjects).*

Acquisition

Imaging type(s)	*Specify: functional, structural, diffusion, perfusion.*
Field strength	*Specify in Tesla*
Sequence & imaging parameters	*Specify the pulse sequence type (gradient echo, spin echo, etc.), imaging type (EPI, spiral, etc.), field of view, matrix size, slice thickness, orientation and TE/TR/flip angle.*
Area of acquisition	*State whether a whole brain scan was used OR define the area of acquisition, describing how the region was determined.*

Diffusion MRI ☐ Used ☐ Not used

Preprocessing

Preprocessing software	*Provide detail on software version and revision number and on specific parameters (model/functions, brain extraction, segmentation, smoothing kernel size, etc.).*
Normalization	*If data were normalized/standardized, describe the approach(es): specify linear or non-linear and define image types used for transformation OR indicate that data were not normalized and explain rationale for lack of normalization.*
Normalization template	*Describe the template used for normalization/transformation, specifying subject space or group standardized space (e.g. original Talairach, MNI305, ICBM152) OR indicate that the data were not normalized.*
Noise and artifact removal	*Describe your procedure(s) for artifact and structured noise removal, specifying motion parameters, tissue signals and physiological signals (heart rate, respiration).*
Volume censoring	*Define your software and/or method and criteria for volume censoring, and state the extent of such censoring.*

Statistical modeling & inference

Model type and settings	*Specify type (mass univariate, multivariate, RSA, predictive, etc.) and describe essential details of the model at the first and second levels (e.g. fixed, random or mixed effects; drift or auto-correlation).*
Effect(s) tested	*Define precise effect in terms of the task or stimulus conditions instead of psychological concepts and indicate whether ANOVA or factorial designs were used.*

Specify type of analysis: ☐ Whole brain ☐ ROI-based ☐ Both

Statistic type for inference<br>(See Eklund et al. 2016)	*Specify voxel-wise or cluster-wise and report all relevant parameters for cluster-wise methods.*
Correction	*Describe the type of correction and how it is obtained for multiple comparisons (e.g. FWE, FDR, permutation or Monte Carlo).*

Models & analysis

n/a	Involved in the study
☐	☐ Functional and/or effective connectivity
☐	☐ Graph analysis
☐	☐ Multivariate modeling or predictive analysis

Functional and/or effective connectivity	*Report the measures of dependence used and the model details (e.g. Pearson correlation, partial correlation, mutual information).*
Graph analysis	*Report the dependent variable and connectivity measure, specifying weighted graph or binarized graph, subject- or group-level, and the global and/or node summaries used (e.g. clustering coefficient, efficiency, etc.).*
Multivariate modeling and predictive analysis	*Specify independent variables, features extraction and dimension reduction, model, training and evaluation metrics.*
